# Readability and Suitability of COPD Consumer Information

**DOI:** 10.1155/2017/2945282

**Published:** 2017-08-29

**Authors:** Kathryn Fullmann, David F. Blackburn, Mark E. Fenton, Holly Mansell

**Affiliations:** ^1^College of Pharmacy and Nutrition, University of Saskatchewan, Saskatoon, SK, Canada; ^2^Division of Critical Care and Sleep Medicine, Department of Medicine, University of Saskatchewan, Saskatoon, SK, Canada

## Abstract

**Background:**

Information leaflets have been shown to positively or negatively impact adherence, depending on their content. The objective of this study was to perform an appraisal of the consumer information provided in COPD inhaler monographs.

**Methods:**

COPD inhalers were identified from the Health Canada Drug Product Database. Medication information and instructions for inhaler use were analyzed for readability by seven formulas, with an acceptability threshold of grades 6–8. Three researchers rated suitability using a modified Suitability Assessment of Materials (SAM) tool and assessed leaflets for explicit warnings.

**Results:**

Twenty-six inhalers with a COPD indication were evaluated. Medication information sections were rated as “difficult to read” or “hard,” and 85% (22/26) had a reading level above grade 8. The instructions for inhaler use were rated as “easy” or “fairly easy” to read and 63% (16/26) met the threshold by all formulas. While all leaflets achieved superior suitability ratings, extreme warnings included risk of premature death (*n* = 12), risks of serious injury (*n* = 26), serious interactions (*n* = 26), and statements that convey a serious consequence to therapy (*n* = 26).

**Conclusion:**

While COPD information leaflets in Canada performed well in terms of readability and suitability, overemphasis on side effects, warnings, and precautions may contribute to patient fear and nonadherence.

## 1. Background

Information leaflets are considered an important facet of patient education for all types of prescription medications [1]. In the case of inhaled agents for COPD, printed information not only serves to provide basic education about the drug but also contains detailed instructions about proper technique for administration. Indeed, administration of inhalers requires a certain degree of technical competence and suboptimal technique has been associated with an increased risk of hospitalizations, along with increased use of antibiotics and corticosteroids [2, 3].

In Canada alone, at least $1.5 billion dollars are spent on COPD hospitalizations annually [4], and COPD is the 4th leading cause of death in Canada and the only major chronic health condition where mortality rates are actually increasing [4, 5]. Although many medications were shown to reduce the risk of hospitalization [6], their use is clearly suboptimal. An average of 60% of patients with COPD do not adhere to prescribed therapy and up to 85% of patients use their inhalers ineffectively [7].

Patient information leaflets have the ability to positively enhance medication knowledge, patient satisfaction, and medication adherence [8]. On the other hand, poorly presented information in monographs can lead to misinterpretations of adverse effects and nonadherence to the prescribed therapy [9, 10]. Guidelines have been suggested on how to write patient friendly information appropriate for the general public [11]. Nevertheless, the readability of these materials often exceeds the recommended grade level [12]. Moreover, information leaflets have frequently been criticized for their potential to increase patient fears over drug safety [10].

The purpose of this study was to conduct an appraisal of the quality of information provided in the product monographs of COPD inhalers available in Canada.

## 2. Methods

### 2.1. COPD Inhalation Devices Identification

A search was conducted to identify all inhaled medications marketed in Canada for COPD using the national Drug Product Database [13]. Search terms were selected within each field of the online database as follows: Status: “marketed and approved”; (i) Class(es): “human”; (ii) Route(s) of administration: “inhalation”; (iii) Dosage form(s): “all”; and (iv) Schedules: “prescription”. Monographs were reviewed to identify devices specifically indicated for the treatment of COPD. Nebulized products were excluded.

For each product monograph identified, the consumer information section written in English (i.e., Part 3) was assessed for readability and suitability. In addition, a subjective assessment was undertaken to identify potential messages that might exaggerate the perception of risk associated with regular use.

### 2.2. Readability

While there are a variety of methods used to assess the readability of written materials, there is no consensus as to which formula is best suited for assessing patient education materials. To improve the validity of the results, it is favourable to use a variety of methods [14]. As such the readability of each leaflet was assessed with seven formulas: the Flesch Reading Ease formula [15], the Flesch-Kincaid Grade Level readability formula [16], the Gunning Fog Index [17], the SMOG Index [18], the Automated Readability Index [19], the Coleman-Liau Index [20], and the Linsear Write Formula [21]. These formulas use average sentence length, number of syllables, or average word length to calculate a reading ease or a reading grade level (Table 1). It is suggested that health information should be written no higher than grades 6–8 reading level to accommodate patients with poor health literacy [11].

Consumer information in each monograph was separated into “medication information” and “instructions on inhaler use.” Each of these sections was entered separately into an online readability calculator [22]. Sections pertaining to reporting side effects to Health Canada and supplemental information were excluded.

### 2.3. Suitability

A suitability assessment was conducted on the leaflets to evaluate additional factors that may impact health literacy [11]. Two different researchers independently assessed each document using a modified version of the Suitability Assessment of Materials (SAM) tool [23, 24]. A third researcher was used to make a final decision in the event of a discrepancy. The modified SAM tool consisted of 16 items after removal of irrelevant elements (“cultural appropriateness”, “summary reviews”, “cover graphics”, “lists/tables”, and “interaction used”) (Table 2). The Flesch-Kincaid Grade Level formula [16] was used to assess item (2)(a): Reading Grade Level.

Each factor was given a rating of “superior,” “adequate,” or “not suitable,” which has a value of 2, 1, or 0, respectively [11, 23]. The values for each inhaler were summed and divided by the maximum score of 32 to produce a percentage. The percentages were categorized as 70–100% (“superior” material), 40–69% (“adequate” material), and 0–39% (“not suitable” material) [11, 23].

### 2.4. Medication Risks

Finally, a subjective review of each monograph was conducted to identify messages that appeared to exaggerate the risk posed to patients. Messages were categorized as highlighting (a) risks of premature death; (b) risks of serious injury; (c) risks of serious interactions; (d) statements that convey a serious consequence to therapy.

## 3. Results

One hundred and fifty-one medications met the search criteria on the Drug Product Database. After excluding the nebulized medications (*n* = 40), 26 inhalers were found to have a COPD indication and were assessed for readability and suitability (Table 3). Of these, 22 were branded products and four were produced by generic companies.

### 3.1. Readability

Readability of the medication information sections from each of the 26 leaflets was rated as “fairly difficult,” “difficult,” or “hard.” None of the monographs were assessed within the recommended grade level of 6–8 by all formulas, and only 26% (7/26) met the target by at least one method (Table 4). In contrast, sections relating to inhaler use instructions were rated as “easy” or “fairly easy” to read and the most complex monographs were rated as “average.” The majority of the instructions for inhaler use (77%, 20/26) met the target grade level of 6–8 by all formulas. (Table 5).

### 3.2. Suitability

All patient information documents achieved a “superior” rating in the categories of purpose, content topics, scope, sentence construction, and behaviors but were deemed “not suitable” for their suboptimal reading grade level. The number of graphics in each leaflet ranged from 3 to 14, with a mean number of 8.5. Nevertheless, all documents received “superior” ratings for containing applicable information on what the medication was for and how to use the devices.

### 3.3. Medication Safety

None of the monographs contained a section dedicated to the benefits of the medication but all contained explicit information on potential risks. Twelve of the 26 inhalers (46%) contained a highlighted warning about the risk of asthma-related deaths associated with the use of the product (i.e., all products containing long-acting beta-2 agonists). All products (26/26) contained risks of serious injury, serious interactions, and statements that convey a serious consequence to therapy. For instance, warnings about dizziness or blurred vision were frequently present and included cautions about driving or operating machinery (*n* = 11). All leaflets had warnings on excessive use, using strong language such as* “extremely dangerous,”* or to* “contact a health care provider immediately if you think you have taken too much.”* A statement indicating that* “the safety and effectiveness in children under the age of 4 years is not known”* was found on all salbutamol inhalers (*n* = 6) (and in one leaflet the “safe age” was listed at 6). Some of the warnings are not supported in the literature, such as advising consumers to avoid contact with anyone with measles or the chickenpox, in the case of inhaled corticosteroids (*n* = 3).

In general, warnings, precautions, and drug interactions were a major focus of the information provided for each product, often taking up the majority of the space in the leaflet.

## 4. Discussion

The provision of patient education is a critical factor for the success of inhaler medications in patients with COPD [25]. Although written information is no substitute for face-to-face education, it has an undeniable influence on the use and perceptions of medications [25]. Health Canada mandates that drug manufacturers develop a detailed consumer information leaflet to educate patients on each medication's purpose, proper administration, and potential side effects [26]. The document should be written using the “simplest, shortest words possible,” using a language and format that is appropriate for the general public [27].

In general, COPD inhaler monographs contained highly readable instructions for use while more complex language was found in their patient information sections. Achieving an acceptable reading level is important, since approximately 60% of Canadians over age 16 have a low level of health literacy and lack the necessary skills to properly manage their health independently [28]. Poor health literacy in COPD is associated with increased disease severity, frequent hospitalizations, and a decrease in quality of life [29]. Low education levels are also a predictor of poor or incorrect inhaler technique [3, 30]. Improved outcomes in COPD will not be achievable if patients cannot understand how to use the devices. Although we find it encouraging that the instructions for inhaler use were generally rated as acceptable, there is certainly room for improvement with respect to patient information.

The suitability of COPD inhaler monographs was rated as “superior” for the applicability of information and how to use the devices. Nevertheless, all of the leaflets dedicated substantial space to the issue of medication safety, including explicit warnings about the risk of premature death. In many cases, more space was dedicated to warnings than correct administration technique. Not one of the leaflets we analyzed had a section dedicated to benefits. Including such information is arguably as important [31], since patients often do not recall all of the information provided by their health care provider regarding the rationale for use [32].

It has previously been suggested that written sources of drug information* “seem designed more to satisfy [governments and liability lawyers than] to teach patients*…*”* [33]. In our study, all monographs for inhalers containing long-acting beta-agonists (*n* = 12) warned about a risk of premature death. A recent systematic review of randomized studies in adult asthma patients found 46 deaths in 17,572 patients randomized to salmeterol or formoterol (0.26%) compared to 33 deaths in 16,380 patients randomized to control (0.20%) for a pooled OR of 1.37 (95% CI: 0.88 to 2.13) [34]. Although it must be acknowledged that signals of risk need to be clearly communicated to patients, the tone and severity of the monographs examined in this study far exceeded the objective risks identified in clinical trials [34]. Furthermore, in the treatment of COPD, LABAs used as monotherapy are recognized as safe and effective therapeutic options [6].

Several other examples of excessive warnings were discovered as well. Despite the fact that inhaled short-acting beta-agonists are widely used as first-line therapy for the treatment of bronchospasm in pediatric patients [35, 36], one of the first warnings on salbutamol leaflets was a statement indicating that* “the safety and effectiveness in children under the age of 4 years is not known.”* Leaflets for anticholinergics warned patients to exercise caution when operating machinery or avoid driving due to the risk of dizziness or blurred vision. The risk of systemic absorption from inhaled anticholinergics is negligible [37], and these adverse effects have been reported at an incidence of 3% or less (which in most cases was similar to placebo) [37–41]. Furthermore, we are unaware of any studies that have tested the effects of inhaled anticholinergics on the ability to drive.

Even more alarming is the fact that several of the warnings in the monographs are not consistent with current literature. For instance, all inhaled corticosteroids advised consumers to avoid contact with individuals with measles or chicken pox. While the risk of acquiring these illnesses is increased in immunocompromised individuals taking oral corticosteroids, this does not hold true for inhaled formulations [42].

Several studies have indicated that negative information may cause fear and provoke nonadherence to medications. In one study in Australia, a third of consumers (35%, 242/691) indicated they had concerns after reading the consumer medication information [43], while in another study in Copenhagen 32% of patients (35/111) stated that they had stopped taking medication due to information about adverse effects [44]. Recently, a qualitative study (*n* = 35) discovered that the vast amount of side effects and drug interactions reported in package leaflets of commonly prescribed medications provoked fear and anxiety and caused patients to stop their medication or alter their dosage regimen without consulting a health care provider [10].

Studies have shown that confidence in drug therapy could be low among patients with COPD [45], and medication underuse is the most prevalent type of nonadherence with this illness [46]. Rather than emphasizing the (often unfounded) risks, manufacturers should aim to encourage adherence by describing the medication's potential benefits. Studies have demonstrated that patients want information that is encouraging to read and how much a medicine will assist them [10, 47]. We realize that drug manufacturers have a duty to warn patients about foreseeable side effects of medications but argue that the risk/benefit approach should be used. Product warnings should only be included in the leaflet, if it is reasonable and substantial enough to warrant concern. Furthermore, manufacturers should make an effort to consult with the ultimate stakeholder—the consumer—prior to publishing the product monographs.

There are several limitations that should be acknowledged with this study. First, only Canadian monographs written in English were included in this evaluation. The patient medication information sections were retrieved from the Health Canada Drug Product Database, so we were unable to assess the layout of the information that is dispensed with the devices. (Health Canada archives the monographs in pdf. format, whereas the information provided with the devices may be in the format of a booklet.) Readability and suitability tests were used as surrogate markers to evaluate the likelihood of patient comprehension of the materials, and a subjective assessment was used to identify explicit warnings. A study involving actual patients would be needed to directly assess patient comprehension and to truly understand the perceptions and consequences associated with the information presented in the leaflets.

## 5. Conclusion

Consumer information intended to educate patients on medications for COPD provides adequate instructions for use; however, the main messages contained in the documents were negative, severe, and sometimes inappropriate. In addition, improvements in the readability of the patient information sections are possible since none of them were rated at the appropriate reading level. Previous calls to improve the design of educational materials have not been acted upon.

## Figures and Tables

**Table 1:** Readability interpretation.

(i) Flesch Reading Ease	(i) Gunning Fog	(i) Flesch-Kincaid Grade Level (ii) Coleman-Liau Index (iii) SMOG Index (iv) Automated Readability Index (v) Linsear Write Formula
90–100: very easy	5: readable 10: hard 15: difficult 20: very difficult	Number is considered equivalent to grade level. Anything above 12 is considered college level.
80–89: easy		
70–79: fairly easy		
60–69: standard		
50–59: fairly difficult		
30–49: difficult		
0–29: very confusing		

SMOG: simple measure of Gobbledygook.

**Table 2:** Modified Suitability Assessment.

Category	Element
(1) Content	(a) Purpose (the purpose is evident) (b) Content topics (suggests behaviors that will help solve a problem) (c) Scope (scope is limited to the purpose of objective)

(2) Literacy demand	(a) Reading grade level (reading grade level score) (b) Writing style (text is written in a conversational style and active voice) (c) Vocabulary (vocabulary uses common words) (d) Sentence construction (context is given first) (e) Advanced organizers (advanced organizers are used to tell what is next)

(3) Graphics	(a) Type of illustrations (graphics are appropriate for the communication) (b) Relevance of illustrations (illustrations provide key ideas and messages) (c) Graphic captions (graphics have a corresponding caption)

(4) Layout and type	(a) Layout (layout factors) (b) Typography (font size and style) (c) Subheading (subheadings are used)

(5) Learning stimulation and motivation	(a) Behavior (behaviors are modeled and specific) (b) Motivation (actions are achievable promoting self-efficacy)

**Table 3:** 

Inhaler	Type	Manufacturer
Advair Diskus	DPI	GlaxoSmithKline
Airomir^*∗*^	MDI	Valeant Canada LP
Anoro Ellipta	DPI	GlaxoSmithKline
Apo-Salvent CFC Free^*∗*^	MDI	Apotex
Atrovent HFA	MDI	Boehringer Ingelheim
Breo Ellipta^a^	DPI	GlaxoSmithKline
Bricanyl Turbuhaler	DPI	AstraZeneca
Combivent Respimat	SMI	Boehringer Ingelheim
Duaklir Genuair	DPI	AstraZeneca
Foradil Aerolizer	DPI	Novartis Pharmaceuticals
Incruse Ellipta	DPI	GlaxoSmithKline
Inspiolto Respimat	SMI	Boehringer Ingelheim
Novo-Salbutamol HFA^*∗*^	MDI	Teva Canada
Onbrez Breezhaler	DPI	Novartis Pharmaceuticals
Salbutamol HFA^*∗*^	MDI	Sanis Health
Seebri Breezhaler	DPI	Novartis Pharmaceuticals
Serevent Diskhaler Disk	DPI	GlaxoSmithKline
Serevent Diskus	DPI	GlaxoSmithKline
Spiriva	DPI (handihaler)	Boehringer Ingelheim
Spiriva Respimat	SMI	Boehringer Ingelheim
Striverdi Respimat^b^	SMI	Boehringer Ingelheim
Symbicort Turbuhaler^b^	DPI	AstraZeneca
Tudorza Genuair	DPI	AstraZeneca
Ultibro Breezhaler	DPI	Novartis Pharmaceuticals
Ventolin Diskus	DPI	GlaxoSmithKline
Ventolin HFA	HFA	GlaxoSmithKline

^**∗**^Generic products; ^a^only 100/25 mcg/dose indicated in COPD; ^b^approved by Health Canada but not currently marketed; MDI: metered dose inhaler; DPI: dry powdered inhaler; SMI: soft mist inhaler.

**Table 4:** Readability of patient medication information.

Inhaler	Flesch Reading Ease	Gunning Fog	Flesch-Kincaid Grade Level	Coleman-Liau Index	SMOG Index	Automated Readability Index	Linsear Write Formula
Advair Diskus	58.9	11.2	8.5	11	8.3	8.4	8
Airomir	44.8	14.2	12.5	11	12	12.5	15.4
Anoro Ellipta	54.1	11	9.6	10	9.6	8.7	10.6
Apo-Salvent CFC Free	48.8	14.1	12.2	10	10.7	12.4	15
Atrovent HFA	42.6	15.6	12.8	12	11.8	13.4	15.1
Breo Ellipta	55.9	11.8	10	10	9.5	10.1	11.8
Bricanyl Turbuhaler	44.3	13.5	11.8	13	10.8	12.3	12.9
Combivent Respimat	30.6	17.3	14.9	14	13.5	15.6	17.3
Duaklir Genuair	35.5	15.7	13.9	13	12.9	14.5	16.2
Foradil Aerolizer	46.4	13	10.8	12	10.2	10.5	11.1
Incruse Ellipta	58.2	10.7	8.9	10	8.6	8.4	8.7
Inspiolto Respimat	33.1	15.4	13.9	13	12.5	14.2	15.3
Novo-Salbutamol HFA	45.2	13.7	12.1	11	11	12.2	13.8
Onbrez Breezhaler	51.3	11.7	10.1	13	8.8	11	9.4
Salbutamol HFA	50	13.2	11.4	11	10.5	11.6	13.7
Seebri Breezhaler	49.1	12.5	11	12	9.1	11.9	11.6
Serevent Diskhaler Disk	58.7	10.7	8.5	11	8.4	8.7	8
Serevent Diskus	58.1	10.9	8.6	11	8.5	8.4	8.1
Spiriva	46.5	13.4	11.5	12	10.9	11.8	13.1
Spiriva Respimat	42.3	14.2	12.4	12	11.6	12.5	14.1
Striverdi Respimat	41.1	13.9	12.3	13	11.7	12.9	13.6
Symbicort	58.9	9.8	8.6	10	8.6	8.1	8.3
Tudorza Genuair	47.5	13.5	11	12	10.8	11	12.3
Ultibro Breezhaler	45.6	12.7	11.4	13	10.1	12.1	12.1
Ventolin Diskus	45.1	14.3	12.9	11	11.2	13.7	15.7
Ventolin HFA	51.4	13.2	11.3	10	10.5	11.5	13.7

**Table 5:** Readability of inhaler use instructions.

Inhaler	Flesch Reading Ease	Gunning Fog	Flesch-Kincaid Grade Level	Coleman-Liau Index	SMOG Index	Automated Readability Index	Linsear Write Formula
Advair Diskus	81.6	6.1	4.2	7	4.6	3.2	4.3
Airomir	80.8	7.5	5.4	7	6.2	5.2	6.9
Anoro Ellipta	74.3	7.3	5.5	7	6.7	3.9	5.6
Apo-Salvent CFC Free	81.2	7.6	5.4	7	5.9	5.2	7
Atrovent HFA	66.8	10.7	7.4	9	8.4	7	8
Breo Ellipta	77.8	7.5	5	7	6.5	3.5	5.5
Bricanyl Turbuhaler	74.8	8.6	6.4	7	7	5.9	7.5
Combivent Respimat	80.3	6.8	4.8	7	5.9	3.8	5.6
Duaklir Genuair	65.8	9.8	8.3	9	8.4	8.7	10.5
Foradil Aerolizer	79	7.1	4.7	8	5.4	4.4	4.8
Incruse Ellipta	74.9	7.5	5.5	7	6.8	3.9	5.6
Inspiolto Respimat	79.4	6.8	5	7	5.8	3.8	5.5
Novo-Salbutamol HFA	71.7	9.8	6.6	8	7.5	6	7.4
Onbrez Breezhaler	69.8	7.2	6.2	10	5.8	6.3	5.4
Salbutamol HFA	72.8	8.8	6.2	9	6.9	5.8	6.6
Seebri Breezhaler	73.6	6.7	5.5	9	5.1	5.4	4.7
Serevent Diskhaler Disk	81.2	6.5	4.8	8	4.9	4.8	5.4
Serevent Diskus	82.6	5.7	4	7	4.4	2.8	4
Spiriva Handihaler	79.9	5.7	4.4	8	5.3	4	4.4
Spiriva Respimat	80.4	6.7	4.8	7	5.8	3.6	5.5
Striverdi Respimat	81.2	6.5	4.6	7	5.7	3.6	5.2
Symbicort	82.5	6.9	4.3	6	5.7	3	5
Tudorza Genuair	64.1	10.3	8.6	10	9	9.1	11
Ultibro Breezhaler	71.1	6.7	5.7	10	5.8	5.5	4.9
Ventolin Diskus	80.4	5.8	4.6	7	5.6	3.5	5
Ventolin HFA	73.8	8.8	6.1	8	6.9	5.7	6.6

## References

[1] Yamamoto M. (2015). Patient drug information leaflets for risk/benefit communication. *Journal of Pharmacovigilance*.

[2] Sanchis J., Gich I., Pedersen S. (2016). Systematic Review of Errors in Inhaler Use: Has Patient Technique Improved Over Time?. *Chest*.

[3] Melani A. S., Bonavia M., Cilenti V. (2011). Inhaler mishandling remains common in real life and is associated with reduced disease control. *Respiratory Medicine*.

[4] Mittmann N., Kuramoto L., Seung S. J., Haddon J. M., Bradley-Kennedy C., FitzGerald J. M. (2008). The cost of moderate and severe COPD exacerbations to the Canadian healthcare system. *Respiratory Medicine*.

[5] Benady S. (2010). *THe Human And Economic Burden of COPD: A Leading Cause of Hospital Admission in Canada*.

[6] *Global Initiative for Chronic Obstructive Lung Disease Global Strategy for the Diagnosis*.

[7] Restrepo R. D., Alvarez M. T., Wittnebel L. D. (2008). Medication adherence issues in patients treated for COPD. *International Journal of Chronic Obstructive Pulmonary Disease*.

[8] Sustersic M., Gauchet A., Foote A., Bosson J.-L. (2016). How best to use and evaluate Patient Information Leaflets given during a consultation: A systematic review of literature reviews. *Health Expectations*.

[9] Carrigan N., Raynor D. K., Knapp P. (2008). Adequacy of patient information on adverse effects: An assessment of patient information leaflets in the UK. *Drug Safety*.

[10] Herber O. R., Gies V., Schwappach D., Thürmann P., Wilm S. (2014). Patient information leaflets: Informing or frightening? A focus group study exploring patients' emotional reactions and subsequent behavior towards package leaflets of commonly prescribed medications in family practices. *BMC Family Practice*.

[11] (2002). *National Literacy and Health Program Good Medicine for Seniors: Guidelines for Plain Language and Good Design in Prescription Medication*.

[12] Pires C., Vigário M., Cavaco A. (2015). Readability of medicinal package leaflets: A systematic review. *Revista de Saude Publica*.

[13] Health Canada Drug and Health Products. Drug Product Database. Available at: http://www.hc-sc.gc.ca/dhp-mps/prodpharma/databasdon/index-eng.php

[14] Badarudeen S., Sabharwal S. (468). Assessing readability of patient education materials: current role in orthopaedics clin orthop relat res. *Clinical Orthopaedics and Related Research*.

[15] Flesch R. (1948). A new readability yardstick. *Journal of Applied Psychology*.

[16] Kincaid J. P., Fishburn R. P., Rogers R. L., Chissom B. S. (1975). Derivation of new readability formulas (automated readability index, fog count, and flesch reading ease formula) for navy enlisted personnel. *Institute for Simulation and Training*.

[17] Gunning R. (1952). *The Technique of Clear Writing*.

[18] McLaughlin H. G. (1969). SMOG Grading: a new readability formula. *J Read*.

[19] Smith E. A., Senter R. J. (1967). Automated readability index. *AMRL-TR Journal*.

[20] Coleman M., Liau T. L. (1975). A computer readability formula designed for machine scoring. *Journal of Applied Psychology*.

[21] Eltorai A. E. M., Naqvi S. S., Ghanian S. (2015). Readability of Invasive Procedure Consent Forms. *Clinical and Translational Science*.

[22] Readability Formulas. Available at: http://www.readabilityformulas.com. Accessed: 25 January 2017

[23] Doak C., Doak L. G., Root J. H., Belcher M. (1996). Assessing suitability of materials. *Teaching Patients with Low Literacy Skills*.

[24] LeBrun M., DiMuzio J., Beauchamp B., Reid S., Hogan V. (2013). Evaluating the health literacy burden of canada’s public advisories: a comparative effectiveness study on clarity and readability drug saf. *Drug Safety*.

[25] Lareau S. C., Hodder R. (2012). Teaching inhaler use in chronic obstructive pulmonary disease patients. *Journal of the American Academy of Nurse Practitioners*.

[26] Health Canada Drug and Health Products. Frequently Asked Questions: Product Monographs Posted to the Health Canada Website. 2014. Available at: http://www.hc-sc.gc.ca/dhp-mps/prodpharma/applic-demande/guide-ld/monograph/pm_qa_mp_qr-eng.php. Accessed: 7 February 2017

[27] Health Canada Drug and Health Products. Guidance document: product monograph. (2013). Available at: http://www.hc-sc.gc.ca/dhp-mps/prodpharma/applic-demande/guide-ld/monograph/pm_mp_2013-eng.php#a110. Accessed: 7 Feb 2017

[28] (2008). *Health Literacy in Canada: A Healthy Understanding*.

[29] Omachi T. A., Sarkar U., Yelin E. H., Blanc P. D., Katz P. P. (2013). Lower health literacy is associated with poorer health status and outcomes in chronic obstructive pulmonary disease. *Journal of General Internal Medicine*.

[30] Pothirat C., Chaiwong W., Phetsuk N., Pisalthanapuna S., Chetsadaphan N., Choomuang W. (2015). E valuating inhaler use technique in COPD patients. *International Journal of COPD*.

[31] Dickinson R., Raynor D. K., Knapp P., MacDonald J. (2016). Providing additional information about the benefits of statins in a leaflet for patients with coronary heart disease: A qualitative study of the impact on attitudes and beliefs. *BMJ Open*.

[32] Kessels R. P. C. (2003). Patients' memory for medical information. *Journal of the Royal Society of Medicine*.

[33] Avorn J., Shrank W. H. (2009). Communicating drug benefits and risks effectively: There must be a better way. *Annals of Internal Medicine*.

[34] Cates C. J., Wieland L. S., Oleszczuk M., Kew K. M. (2014). Safety of regular formoterol or salmeterol in adults with asthma: an overview of Cochrane reviews. *The Cochrane Database of Systematic Reviews*.

[35] Ducharme F. M., Dell S. D., Radhakrishnan D. (2015). Diagnosis and management of asthma in preschoolers: A Canadian Thoracic Society and Canadian Paediatric Society position paper. *Paediatr Child Health*.

[36] Global Initiative for Asthma (GINA) Global Strategy for Asthma Management and Prevention. [Updated 2016]. Available at: http://www.ginasthma.org; Accessed 28 February 2017

[37] Scullion J. E. (2007). The development of anticholinergics in the management of COPD. *International Journal of COPD*.

[38] (February 2016). *Atrovent HFA (Ipratropium) [Product Monograph]*.

[39] (January 2016). *Spiriva HandiHaler (Tiotropium Bromide) [Prescribing Information]*.

[40] (March 2016). *Incruse Ellipta (Umeclidinium) [Product Monograph]*.

[41] (January 2016). *Seebri Breezhaler (Glycopyrronium) [Product Monograph]*.

[42] Welch M. J. (1994). Inhaled steroids and severe viral infections. *Journal of Asthma*.

[43] Hamrosi K. K., Raynor D. K., Aslani P. (2014). Pharmacist, general practitioner and consumer use of written medicine information in Australia: Are they on the same page?. *Research in Social and Administrative Pharmacy*.

[44] Horwitz A., Reuther L., Andersen S. E. (2009). Patient information leaflets seen through the eyes of patients in a general practice. *Ugeskr Laeger*.

[45] George J., Kong D. C. M., Thoman R., Stewart K. (2005). Factors associated with medication nonadherence in patients with COPD. *Chest*.

[46] Harrow B. S., Strom B. L., Gans J. A. (1997). Impact of pharmaceutical under-utilization: a study of insurance drug claims data. *Journal of the American Pharmacists Association*.

[47] Young A., Tordoff J., Smith A. (2016). "What do patients want?" Tailoring medicines information to meet patients' needs. *Research in Social and Administrative Pharmacy*.

